# Developing new strategies for the gradual integration of sensory imagery scripts into mental training: a commentary on Krüger et al. (2022)

**DOI:** 10.1007/s00426-024-02007-x

**Published:** 2024-07-25

**Authors:** Quang Thong Thai, Martin Lotze

**Affiliations:** Functional Imaging Unit, Diagnostic Radiology and Neuroradiology, University Medicine Greifswald, Greifswald, Germany

## Abstract

Our comment emphasizes the multisensory guidance of mental training processes as pointed out by Krüger et al. 2022. Guided multisensory mental training can focus on different aspects of action performance and gradually increase the vividness of the mental construct. The authors previously described an interaction between neural representation, subjective experience, and training gain. To enrich the subjective experience of the mental process, the establishment and testing of different guided mental training procedures are discussed.

Multisensory imagery and training with guided imagery have recently gained increasing attention. Before developing new strategies for the gradual integration of sensory imagery scripts into mental training, a sound knowledge of the contribution of the integration of sensory elements to training is required. In addition, the neural representation of different aspects of action imagery might be useful to differentiate the underlaying processes. The authors summarize findings on multiple sensory impressions of an action image. In addition to a description of the neural representation, they also address the question of the vividness and quality of imagery. Their outlook on open questions and future research offers promising ideas for further work in this field.

The authors point out that an exclusive focus only on visual or kinesthetic imagery processes in previous imagery research may be incomplete. In the area of mental training in musicians, it has already been established that auditory imagery content precedes and leads the sensorimotor aspects. The authors present a number of publications on instrumentalists and singers that emphasize the importance of auditory imagery (melody, rhythm, intensity and expression).

Especially for laypeople, the instructions for imaging a certain action are diffuse and the vividness of the imagination is low if an internal construct of the action has only been rudimentarily developed. Guided multisensory mental training can focus on different aspects of the execution of the action and increase the vividness of the mental construct step by step. The authors also emphasize the importance of an emotional effect: some imagery scenes are very rewarding, such as their example of swimming in the Caribbean. Integrating environmental aspects not only for the visual domain is an important way of facilitating interactions between the motor and the emotional system to increase motivation and training gain.

The authors have previously shown (Lorey et al., 2011; Zabicki et al., 2019) that the rated intensity of vividness is related to the extent of recruitment of sensorimotor areas, which was measured using functional magnetic resonance imaging (fMRI). Thus, there is an interaction between neural representation, subjective experience and training gain. We found the differentiation of processes involved in action imagery into top down (e.g., instructions), bottom-up (e.g., imagined task), and personal processes (e.g., task performance experience and imagery quality) particularly intriguing. This differentiation could improve the precise understanding of underlying processes for mental practice and help to increase the effectiveness of mental training strategies.

What the authors did not explicitly mention in the top-down modulations is the possibility of first instructing each modality and then having the participants perform an action imagery session in which they focus on only one of the instructed modalities. In the next session they are asked to focus on another instructed modality. After repeating this process with each instructed modality, they might be able to visualize the entire action more vividly. This variable priority training (Gopher et al., 1989) could be a method to further increase the effectiveness of action imagery. This raises the question of whether the order in which the modalities are taught is important and whether it influences the effectiveness of action imagery.

With regard to individual differences in imagery ability, the influence of handedness must be taken into account. A recent study investigated the effect of handedness on motor imagery, showed that left-handers performed better on the imagination task regardless of modality (visual or kinaesthetic). The authors of the study discussed whether the difference in the relevance of motor imagery could explain the superior performance of left-handed individuals. For instance, left-handed individuals often have to mentally change the perspective of the perceived scene or object due to the predominantly right-handed environment (Zapała et al., 2021). Further research is needed to differentiate between the imagery performance of left- and right-handed individuals and to determine the extent to which these results apply to action imagery, as it involves more modalities (acoustic, olfactory and gustatory).

Another aspect that could be important, has not been explicitly mentioned here. It is the representation of one’s own body in the interaction with the tools or the environment. The authors address this issue when discussing laparoscopy training with mental strategies, but do not explicitly point out the challenge of this training for multisensory body integration. This is of great importance for adaptation to a vehicle (boat, car, bike, plane), but also for adaptation to a virtual environment with different skins or tools (Riva et al., 2019). Research on the multisensory integration of body, tools and environment and the role of the parietal lobe and feedforward mechanisms in the cerebellum could advance the field in the coming years and significantly expand our understanding of mental training.

## Data Availability

Not applicable.
